# ECDD-S16 targets vacuolar ATPase: A potential inhibitor compound for pyroptosis-induced inflammation

**DOI:** 10.1371/journal.pone.0292340

**Published:** 2023-11-27

**Authors:** Peeraya Ekchariyawat, Rattatammanoon Saengfak, Sucharat Sanongkiet, Thanapon Charoenwongpaiboon, Suphasuta Khongpraphan, Supaporn Mala, Chularat Luangjindarat, Bumrung Munyoo, Napason Chabang, Sitthivut Charoensutthivarakul, Suparerk Borwornpinyo, Patoomratana Tuchinda, Marisa Ponpuak, Matsayapan Pudla, Pongsak Utaisincharoen

**Affiliations:** 1 Department of Microbiology, Faculty of Public Health, Mahidol University, Bangkok, Thailand; 2 Department of Microbiology, Faculty of Science, Mahidol University, Bangkok, Thailand; 3 Department of Chemistry, Faculty of Science, Silpakorn University, Nakhon Pathom, Thailand; 4 Research Office, Faculty of Dentistry, Mahidol University, Bangkok, Thailand; 5 Excellence Center for Drug Discovery (ECDD), Faculty of Science, Mahidol University, Bangkok, Thailand; 6 School of Bioinnovation and Bio-Based Product Intelligence, Faculty of Science, Mahidol University, Bangkok, Thailand; 7 Center for Neuroscience, Faculty of Science, Mahidol University, Bangkok, Thailand; 8 Department of Biotechnology, Faculty of Science, Mahidol University, Bangkok, Thailand; 9 Department of Oral Microbiology, Faculty of Dentistry, Mahidol University, Bangkok, Thailand; Shantou University Medical College, CHINA

## Abstract

**Background:**

Cleistanthin A (CA), extracted from *Phyllanthus taxodiifolius* Beille, was previously reported as a potential V-ATPase inhibitor relevant to cancer cell survival. In the present study, ECDD-S16, a derivative of cleistanthin A, was investigated and found to interfere with pyroptosis induction via V-ATPase inhibition.

**Objective:**

This study examined the ability of ECDD-S16 to inhibit endolysosome acidification leading to the attenuation of pyroptosis in Raw264.7 macrophages activated by both surface and endosomal TLR ligands.

**Methods:**

To elucidate the activity of ECDD-S16 on pyroptosis-induced inflammation, Raw264.7 cells were pretreated with the compound before stimulation with surface and endosomal TLR ligands. The release of lactate dehydrogenase (LDH) was determined by LDH assay. Additionally, the production of cytokines and the expression of pyroptosis markers were examined by ELISA and immunoblotting. Moreover, molecular docking was performed to demonstrate the binding of ECDD-S16 to the vacuolar (V-)ATPase.

**Results:**

This study showed that ECDD-S16 could inhibit pyroptosis in Raw264.7 cells activated with surface and endosomal TLR ligands. The attenuation of pyroptosis by ECDD-S16 was due to the impairment of endosome acidification, which also led to decreased Reactive Oxygen Species (ROS) production. Furthermore, molecular docking also showed the possibility of inhibiting endosome acidification by the binding of ECDD-S16 to the vacuolar (V-)ATPase in the region of V0.

**Conclusion:**

Our findings indicate the potential of ECDD-S16 for inhibiting pyroptosis and prove that vacuolar H^+^ ATPase is essential for pyroptosis induced by TLR ligands.

## Introduction

Pyroptosis, an emerging type of programmed cell death, is associated with the inflammatory response and can be activated by pathogens or their endotoxins, leading to caspase activation, cell swelling, membrane pore formation and rupture, inflammasome activation, as well as the release of cell contents and pro-inflammatory cytokines such as IL-1β and IL-18. This programmed cell death can be regulated via a canonical or non-canonical pathway. In canonical pyroptosis, it is initiated by the binding of pathogen-associated molecular patterns (PAMPs) to the intracellular pattern recognition receptors (PRRs), such as NLRP1b, NLRP3, NLRC4, AIM3, or Pyrin. These complex proteins then bind to pro-caspase-1 via inflammasome adapter ASC, activating caspase-1 [1]. In contrast, non-canonical pyroptosis pathway, pathogen-associated molecular patterns (PAMPs) can directly bind and activate mouse caspase-11 (orthologs to human caspase-4/5) protein [1, 2]. Upon activation, the active caspases can cleave pro IL-1β and IL-18 to become mature IL-1β and IL-18. In addition, activated-caspase can also cleave gasdermin D (GSDMD) into cleaved-GSDMD, which can connect with phosphatidylinositol (PI) on the cells membrane to form plasma membrane pores, leading to cell swelling, rupture, and cell content release including of IL-1β and IL-18 [1].

The inflammatory response begins upon the recognition of the pathogen-associated molecular patterns (PAMPs) by the pattern-recognition receptors, such as Toll-like receptors (TLRs) expressed on the cell surface and endosomal membrane. TLRs can be categorized into those that signal through MyD88 or Toll/IL-1R domain-containing adaptor-inducing IFN-β (TRIF). In addition, TLRs can be categorized based on their location: at the cell surface (TLR1, TLR2, TLR4, TLR5, TLR6, and TLR10) or on intracellular membrane (TLR3, TLR7, TLR8, TLR9, TLR11, TLR12, and TLR13) [3]. However, in all cases, TLRs induce NF-κB signaling and a standard set of inflammatory genes including the cytokines like TNF-α.

Endocytosis of the ligand and receptors complex is necessary for signal transduction by the cell surface TLRs such as TLR2 and TLR4 [4–7]. For example, the endocytosis of ligand-bound TLR4 is required for activating TRIF-mediated signaling. Besides TLR4, TLR2 has been demonstrated to be endocytosed into endosomes before being sent to lysosomes. The signaling pathway for inducing IFN-β via MyD88-dependent IRF1 and IRF7 pathways also relies on the acidity of the endosomes [6]. Acidification of endosomes is also crucial for the signal transduction of the endosomal TLRs. TLR3, TLR7 and TLR9 signalings require receptor proteolysis in the endolysosomes [8] by the lysosomal cathepsins and endopeptidases to cleave the ectodomain of the TLRs [9].

A recent report showed that TLR4/TRIF-mediated type-I-IFN synthesis is crucial for controlling caspase-11 expression and activation [1, 10, 11]. This enzyme can act as a cytosolic endotoxin receptor and plays a role in pyroptosis under conditions such as LPS-stimulated endotoxemia and bacterial sepsis [12]. Mouse caspase-11 is homologous to human caspase-4/5, which have been confirmed to directly sense intracellular LPS derived from Gram-negative bacteria during macrophage inflammatory responses [13]. Recognition of intracellular LPS facilitates the rapid oligomerization of caspase-4/5/11 leading to the cleavage of the pore-forming protein gasdermin D (GSDMD) to GSDMD-NT, which allows its N-terminal domain to associate with membrane lipids and form pores that induce pyroptotic cell death and the release of proinflammatory cytokines, e.g., IL-1β and IL-18 [14, 15]. In addition, Reactive Oxygen Species (ROS) generated by TLRs has been reported to activate GSDMD [16]. Inhibition of ROS attenuated the cleavage of GSDMD, suggesting that GSDMD activation occurs downstream of ROS release.

Recently cleistanthin A was identified as a potential V-ATPase inhibitor leading to the neutralization of the lysosomal pH [17]. Cleistanthin A (CA) [18], a natural aryl naphthalene lignan glycoside [19–21], was derived from *Phyllanthus taxodiifolius* Beille. This shrub belongs to the Euphorbiaceae family and is found in Thailand’s central and Northeastern parts. Additionally, cleistanthin A was previously isolated from *Cleistanthus patulus* and *Cleistanthus collinus* [19–21]. Cleistanthins have garnered scientific interest due to their potential therapeutics for cancer treatment. However, due to limited research and available information, the potential applications of cleistanthin A besides anti-cancer are not well-established. Recently, our group demonstrated that the derivative of this natural compound, ECDD-S27, can inhibit cancer cell survival by inhibiting autophagy via targeting the vacuolar ATPase [22]. Autophagy, a lysosomal-dependent cellular degradation process, has been connected to several diseases, including cancer and bacterial infectious diseases. This study further extended the analysis of other derivatives of cleistanthin A by studying the chemically modified derivative of cleistanthin A, ECDD-S16. This derivative compound was investigated on its biological function in the context of anti-inflammatory involving TLRs. The results show that ECDD-S16 was able to inhibit endolysosome acidification resulting in the attenuation of proinflammation cytokines release, ROS production and pyroptosis. This may offer a potential candidate for drug development in searching for a new natural compound for various inflammatory diseases.

## Materials and methods

### Cell line and culture condition

Raw264.7 macrophages were purchased from the American Type Culture Collection (ATCC, Manassas, USA). They were cultured in Dulbecco’s modified Eagle’s medium (DMEM) (Hyclone, Logan, UT, USA) supplemented with 10% fetal bovine serum (Gibco Labs,Grand Island, NY, USA) at 37°C under a 5% CO_2_ atmosphere. The cell line passage used throughout the experiment was between 12 to 20.

### Chemical reagents and TLR ligands

CpG Class B ODN 1826 for TLR9, imiquimod for TLR7, Pam2CSK4 for TLR2/6, Pam3CSK4 for TLR2/1, Peptidoglycan for TLR2 and Polyinosinic-polycytidylic acid (poly I:C) for TLR3 were purchased from invivogen. Lipopolysaccharide (LPS) (from an *Escherichia coli*) for TLR4, diphenyleneiodonium (DPI), cytochalasin D, chloroquine and 2′,7′-dichlorofluorescein diacetate (DCFH-DA) were purchased from Sigma-Aldrich. For ECDD-S16 synthesis, all reagents and solvents were purchased from commercial suppliers (Sigma-Aldrich, Merck, or Tokyo Chemical Industry (TCI)) and were used without further purification.

### Synthesis of ECDD-S16 and structure characterization

To synthesis ECDD-S16, 4-fluorobenzoic acid (1.5 eq) was dissolved in dichloromethane under nitrogen gas. *N*,*N′*-Dicyclohexylcarbodiimide (DCC) (1.5 eq) was added, stirring the reaction for 5 minutes. CA (1.0 eq) and 4-dimethylaminopyridine (DMAP) (catalytic amount) was then added to the reaction and the mixture was left overnight at room temperature. When completed, the reaction was diluted with ethyl acetate and washed with saturated NH_4_Cl (aq.), water, brine, and dried over MgSO_4_. The organic portion was filtered and concentrated to give a crude product. Purification was performed using flash column chromatography over silica gel to obtain the desired product ECDD-S16 as off-white powder (36% yield). ECDD-S16 (1); ^1^H NMR (500 MHz, CDCl_3_): 8.11 (dd, *J* = 8.8, 5.4 Hz, 2H), 7.56 (d, *J* = 3.6 Hz, 1H), 7.14 (br t, *J* = 8.6 Hz, 2H), 7.01 (*s*, 1H), 6.94 (dd, *J* = 7.9, 1.0 Hz, 1H), 6.80 (dd, *J* = 5.0, 1.4 Hz, 1H), 6.78–6.75 (m, 1H), 6.01 (d, *J* = 0.9 Hz, 1H), 6.04 (d, *J* = 1.4 Hz, 1H), 5.58 (t, *J* = 6.8 Hz, 1H), 5.46 (dd, *J* = 14.5, 1.6 Hz, 1H), 5.38 (dd, *J* = 14.6, 4.3 Hz, 1H), 5.23 (dd, *J* = 6.1, 0.9 Hz, 1H), 4.23 (dd, *J* = 12.1, 3.4 Hz, 1H), 3.88 (s, 3H), 3.77 (s, 3H), 3.60 (s, 3H), 3.54 (s, 3H), 3.44 (dd, *J* = 11.8, 7.0 Hz, 1H). ^13^C NMR (500 MHz, CDCl_3_): 169.5, 166.1 (C-F, ^1^*J*_C-F_ = 253.8 Hz), 164.5, 151.7, 150.2, 147.4, 143.9, 135.6, 132.4 (C-F, ^3^*J*_C-F_ = 8.8 Hz), 130.5, 128.3, 126.4 (C-F, ^4^*J*_C-F_ = 7.5 Hz), 125.6, 123.50, 119.2, 115.9 (C-F, ^2^*J*_C-F_ = 21.3 Hz), 110.6, 108.2, 105.9, 101.2, 100.7, 81.2, 77.9, 72.0, 67.0, 62.7, 60.1, 58.5, 56.0, 55.8. ^19^F NMR (376 MHz, CDCl_3_): -104.0 (s). ESI-MS m/z: 663.1879 [M+H]^+^, (calcd. for C_35_H_32_FO_12_, 663.1878).

### MTT cell viability assay

Briefly, Raw264.7 macrophages (1.75 × 10^4^ cells/well) were seeded onto a flat-bottom 96-well plate (Corning, NY, USA). The overnight cultures were treated with various concentrations of ECDD-S16 for 18 h. DMSO (Sigma-Aldrich) was used as a negative control. The cell viability was determined by MTT assay using the 3-(4,5-dimethylthiazol-2-yl)-2,5-diphenyltetrazolium bromide (MTT; Sigma-Aldrich). The absorbance of the solution was measured at 540 nm using a microplate reader (Ao Microplate reader, Azure biosystems Inc., Model AC3000). The percent cell viability was calculated by using % cell viability = [(Absorbance of treated cells − Absorbance of blank)/(Absorbance of DMSO control cells − Absorbance of blank)] × 100.

### Activation of Raw264.7 macrophages with TLR ligands

Raw264.7 macrophages (2.5 × 10^5^ cells/well) were seeded overnight in a 6-well plate (Corning, NY, USA). The overnight cultures of Raw264.7 cells were pretreated with ECDD-S16 (0.5 μM) for 1 h, cytochalasin D (2 μg/ml) for 2 h or chloroquine (30 μM) for 15 min before activation with Pam2CSK4, Pam3CSK4, Peptidoglycan (PGN) and *E*.*coli* LPS at a concentration of 100 ng/ml, Poly I:C at a concentration of 50 μg/ml, Imiquimod and ODN1826 at a concentration of 1 μg/ml. At 18 h of stimulation, the supernatant from the treated cells were collected for nitric oxide production, LDH release and cytokine assay. In addition, the treated cells were lyzed and the protein expression was determined by immunoblotting.

### Transfection of Raw264.7 macrophages with TLR2 plasmid

TLR2 plasmid (pcDNA3-TLR2-YFP) was a gift from Doug Golenbock (Addgene plasmid # 13016; http://n2t.net/addgene:13016; RRID:Addgene_13016). Raw264.7 cells were transiently transfected with TLR2 plasmid (5 μg) using Nucleofector solution kit V (Amaxa, London, UK) according to the manufacturer’s instructions. After transfection, the cells were transferred into a new flask containing 10 ml complete media and incubated at 37°C followed by a medium change at 6 h of incubation. After 48 h of transfection with TLR2 plasmid, the cells were then used in assays.

### Immunofluorescent staining

TLR2-YFP-transfected Raw264.7 cells (1.5 × 10^5^ cells/well) were seeded on a glass coverslip in a 24-well plate. The overnight cultures were pretreated with ECDD-S16 (0.5 μM) for 1 h before activation with Pam2CSK4 (1 μg/ml). At 1 h of activation, LysoTracker Red (LTR) (Invitrogen, Massachusetts, USA) dye was then added and the incubation was continued for 2 h. At 3 h of activation, the cells were fixed with 4% paraformaldehyde for 5 min and then washed with PBS. TLR2 trafficking in endosomal compartments was analyzed by confocal laser scanning microscopy (CLSM) (Leica Stellaris 5).

### Determination of nitric oxide (NO)

At indicated time point after stimulation with different TLR ligands, the Griess assay determined the NO production as described previously [23]. Briefly, 50 μl of culture supernatant was mixed with an equal volume of Griess reagent. The concentration of NO was determined by measuring the absorbance at 540 nm (Ao Microplate reader, Azure biosystems Inc., Model AC3000) regarding the standard curve using sodium nitrite.

### LDH assay

The release of lactate dehydrogenase (LDH) in the culture supernatants was measured by cytotoxicity assay to determine the level of pyroptosis. At the indicated time intervals, the supernatants of treated cells were collected, and LDH activity was detected using the CytoTox 96^®^ non-radioactive cytotoxicity assay (Promega, Wisconsin, USA) according to the manufacturer’s instructions. The supernatants were applied to each well of the flat-bottom 96-well plate (NUNC^TM^ Brand Product). The 50 μl of reconstituted substrate mix was added and incubated at room temperature in the dark for 20 min. To stop the reaction, 50 μl of stop solution was applied to each well. The LDH measurements recorded absorbance at 490 nm (Ao Microplate reader, Azure biosystems Inc., Model AC3000). For maximum LDH release, untreated cells were added with a 10X lysis solution and performed the LDH detection in the same manner. The percentage of LDH release was calculated followed by the formula: % LDH release = (Experimental LDH release—Spontaneous LDH release/Maximum LDH release) x100.

### Immunoblotting

The treated cells were lyzed in a lysis buffer containing 20 mM Tris, 100 mM NaCl and 1% NP40. The lysates were separated on 10%, 12% and 15% SDS-PAGE gels. Proteins were transferred onto a nitrocellulose membrane (Amersham Biosciences, Amersham, UK). First, the non-specific binding sites on the membrane were blocked with 5% blocking solution (Roche Diagnostics) for 1 h before proteins were allowed to react with specific primary antibodies against caspase-11, GSDMD, cathepsin D (Abcam, Cambridge, UK) and Actin (Merck Millipore, Darmstadt, Germany) at 4°C overnight. Next, the membranes were washed three times with 0.1% PBST and incubated with horseradish peroxidase-conjugated goat anti-rabbit IgG or goat anti-mouse IgG (R&D Systems, Minnesota, USA) for 1 h at room temperature. Thereafter, the membranes were washed four times with 0.1% PBST before a chemiluminescence substrate (Roche Diagnostics, Basel, Switzerland) was added and protein bands were detected by enhanced chemiluminescence.

### Measurement of intracellular reactive oxygen species (ROS) production

The intracellular ROS levels were measured using cell-permeable non-fluorescence probe 2′,7′-Dichlorofluorescein (DCFH-DA) (Sigma-Aldrich). Briefly, the stimulated cells were washed twice with pre-warmed PBS buffer and loaded with 2 μM DCFH-DA detection reagent. After incubation in darkness for another 20 min at 37°C, the cells were washed twice with an ice-cold PBS buffer and measured immediately. The fluorescence representing intracellular ROS levels was measured with CytoFLEX flow cytometer (Beckman Coulter) using CytExpert.

### Determination of TNF-α and IFN-β production

The supernatant from activated cells was collected. The cytokine production levels were measured by mouse TNF-α kit (BD Bioscience, San Diego, USA) and mouse IFN-β kit (R&D Systems) following the manufacturer’s instructions.

### Molecular docking studies

Molecular docking was performed with Autodock vina software [24]. The Cryo-EM structure of V-ATPase derived from *Saccharomyces cerevisiae* was obtained from a protein data bank (Resolution 2.80 Å PDB code: 7PAP). The protonation states of all amino acids at pH 7.4 was forecast by H^++^ server [25]. The structure of ECDD-S16 compound was prepared by Gaussview (https://gaussian.com/dl/g16_c01.enw). The docking results were depicted by using PyMol. 2D diagram of protein-ligand interactions was generated by PlexView (https://playmolecule.com/PlexView/).

### Statistical analysis

All experiments were performed at least three independent times. The results were expressed as mean ± SEM. All data were analyzed by the Prism software (GraphPad) by using a one-way ANOVA test followed by a post-hoc multiple comparison test based on specific experiments. Asterisks indicate statistically significant differences based on p-values: * for *p* < 0.05, ** for *p* < 0.01, *** for *p* < 0.001 and **** for *p* < 0.0001.

## Results

### ECDD-S16 is a derivative of cleistanthin A

**Fig 1:**
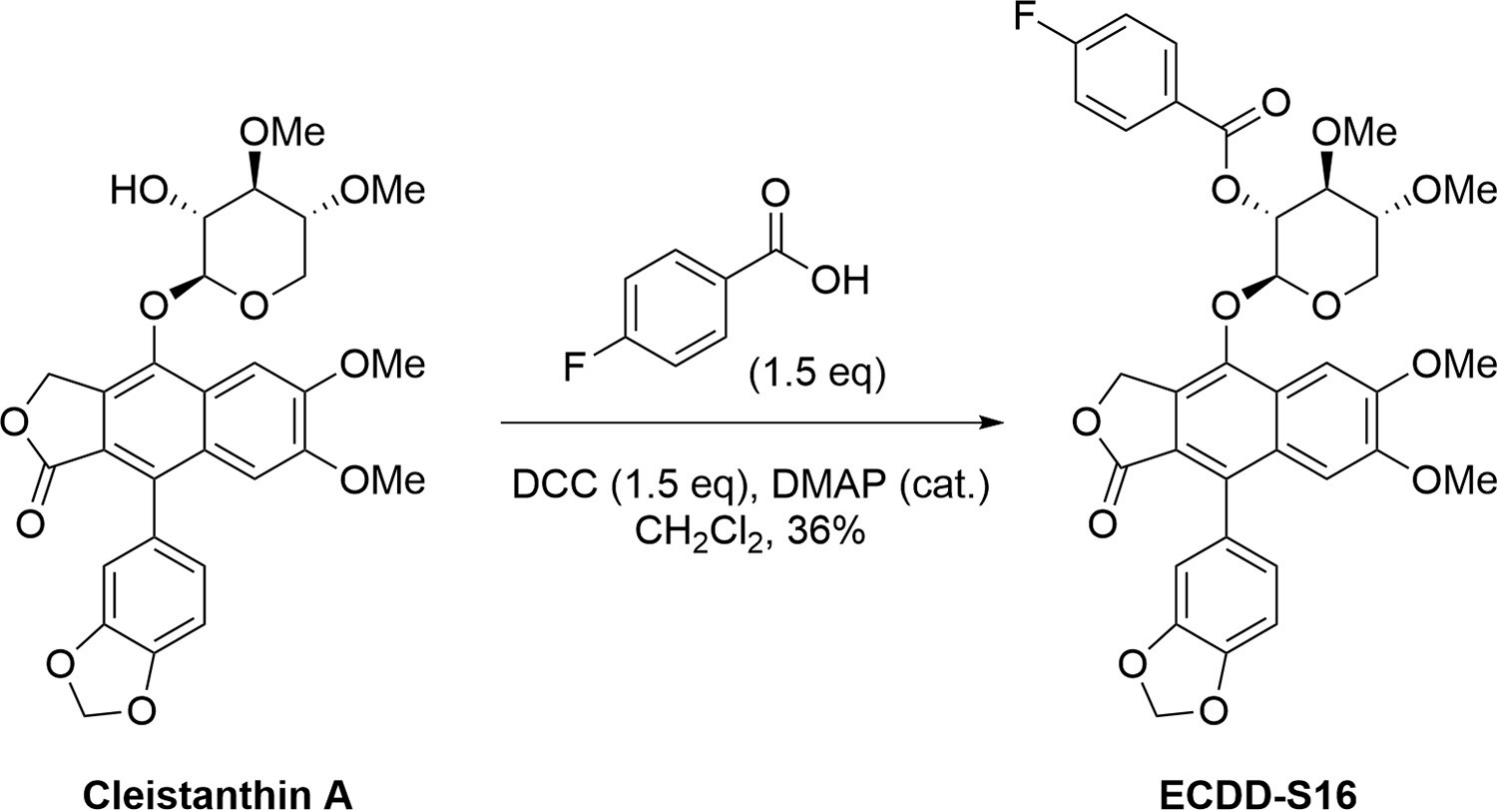
Synthesis of ECDD-S16, a benzoyl ester derivative of cleistanthin. **A.** The esterification reaction between cleistanthin A and 4-fluorobenzoic acid in the presence of dicyclohexylcarbodiimide (DCC) and catalytic 4-dimethylaminopyridine (DMAP) affords ECDD-S16 in 36% yield.

In the present study, ECDD-S16 was successfully prepared from the esterification of cleistanthin A with 4-fluorobenzoic acid in the presence of DCC and catalytic DMAP, resulting in the formation of a new benzoyl ester as shown in Fig 1. Spectroscopic techniques including NMRs and mass spectrometry confirmed the structural integrity of ECDD-S16. Raw264.7 cells were treated with different concentrations of ECDD-S16 and tested for the cytotoxicity of this compound. It should be noted that the viability of Raw264.7 cells treated with this compound at a concentration of 0.5 μM was greater than 80% and the IC_50_ is more than 10 μM.

### Inhibition of proinflammatory cytokine and NO production in TLR-ligand-induced Raw264.7 macrophages by ECDD-S16

**Fig 2:**
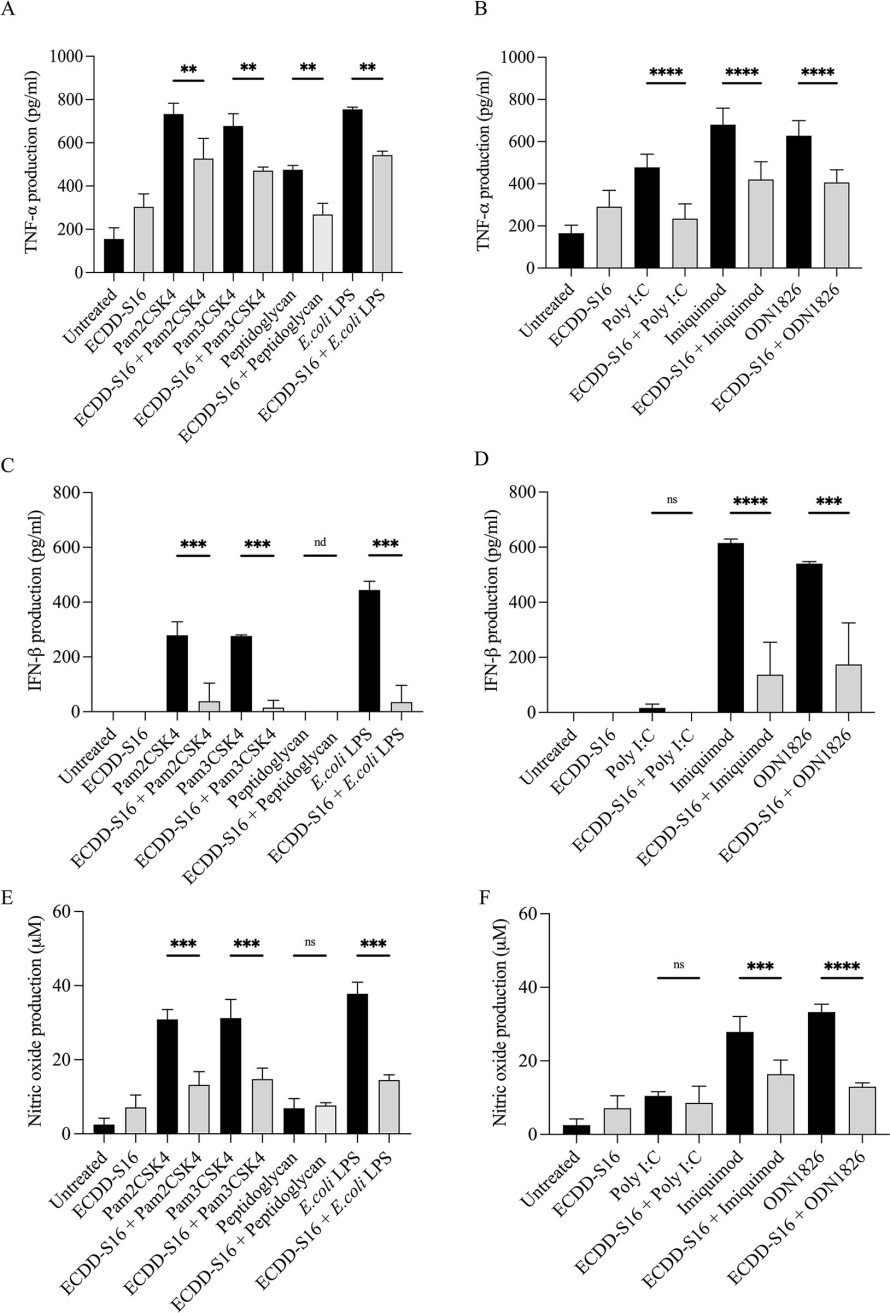
Effect of ECDD-S16 on TLR ligand-induced proinflammatory cytokine and NO production. Raw264.7 cells were pretreated with ECDD-S16 (0.5 μM) for 1 h before stimulation with TLR ligands for 18 h. (A-D) The supernatants were collected and cytokine production was measured by ELISA. (E-F) NO production was determined by Griess assay. Data are expressed as mean ± SEM of three independent experiments. All data were determined by one-way ANOVA followed by Tukey’s multiple comparison test. **p < 0.01, ***p < 0.001,**** *P <* 0.0001. nd = not detected.

The amounts of secreted TNF-α, IFN-β, and NO production were measured to determine whether ECDD-S16 can inhibit the production of proinflammatory cytokines in mouse macrophages in response to TLR ligands. The results showed that ECDD-S16 substantially reduced the levels of TNF-α activated by both surface (Fig 2A) and endosomal TLR ligands (Fig 2B). In addition, IFN-β and NO production was also observed in cells treated by most of the TLR ligands, except for that treated by peptidoglycan and poly I:C (Fig 2C–2F). These findings showed that ECDD-S16 could inhibit the production of inflammatory cytokines and NO in Raw264.7 cells activated with surface and endosomal TLR ligands. It should be noted that ECDD-S16 treatment alone had little to no impact on cytokine secretion in Raw264.7 cells.

### ECDD-S16 inhibits pyroptosis in Raw 264.7 macrophages stimulated with TLR ligands

**Fig 3:**
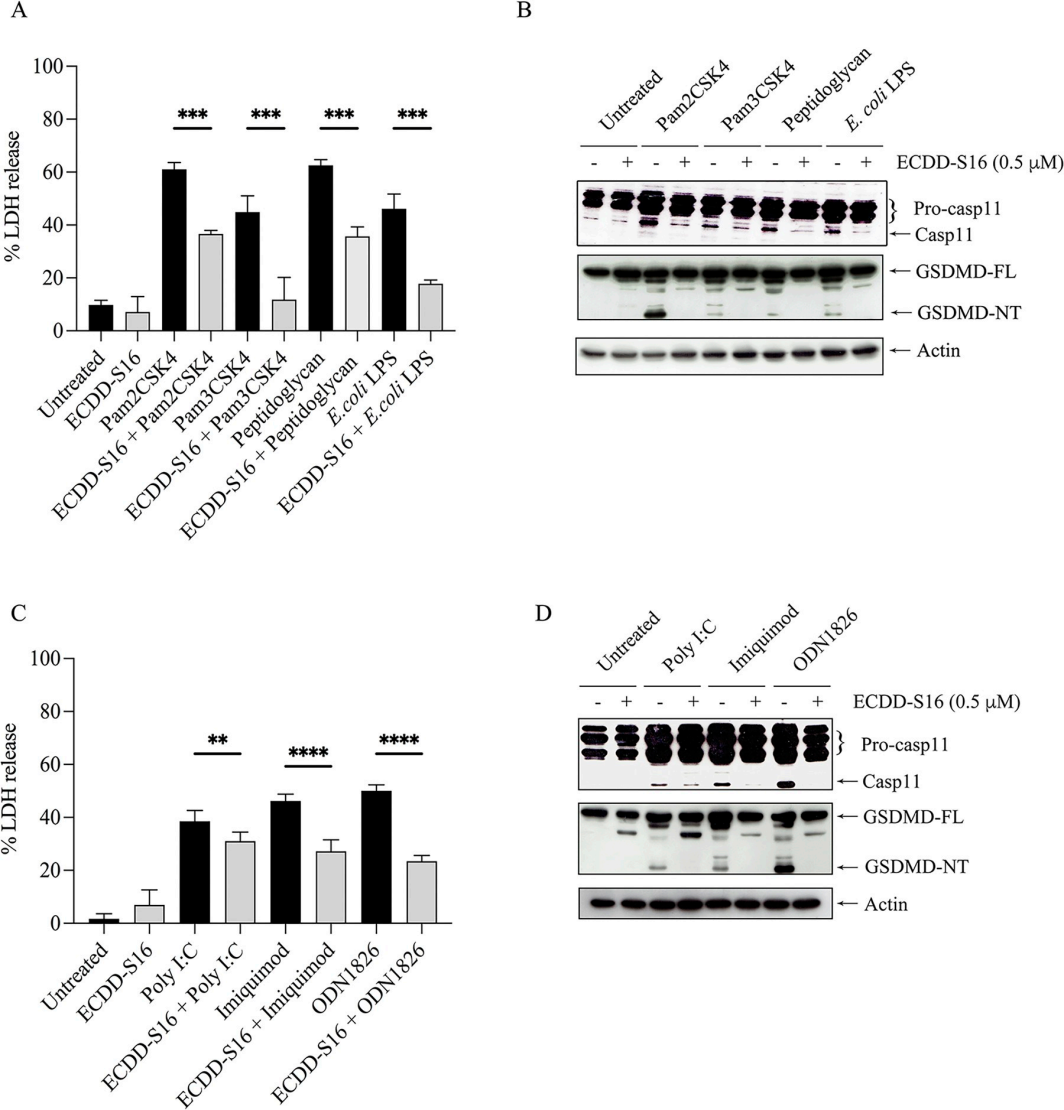
ECDD-S16 inhibits pyroptosis induced by TLR ligands in Raw264.7 cells. Raw264.7 cells were pretreated with ECDD-S16 (0.5 μM) for 1 h before stimulation with TLR ligands for 18 h. (A, C) At the indicated time, the cultured supernatant of treated cells was collected and subjected to analysis by the LDH assay. Data are means ± SEM from at least three independent experiments. All data were determined by one-way ANOVA followed by Tukey’s multiple comparison test. **p < 0.01, ***p < 0.001, ****p < 0.0001. (B, D) The cell lysates were prepared and analyzed by immunoblotting. Representative bands are shown from three independent experiments.

TLR activation induces proinflammatory factor release and activates the inflammasome to promote caspase-1/4/5/11 catalytic activity [13]. In murine macrophages, caspase-11 plays a role as an inflammasome-associated caspase. Caspase-11 cleaves GSDMD to produce an N-terminal fragment which is a crucial determinant for the proinflammatory cell death, known as pyroptosis [13]. As shown in Fig 3A and 3C, the release of LDH from TLR-ligand-treated cells, was significantly impaired when the cells were pretreated with ECDD-S16. These results were consistent with the attenuation of caspase-11 activation and the cleavage of GSDMD-NT (Fig 3B and 3D) suggesting that ECDD-S16 could inhibit pyroptosis induced by these TLR ligands.

### Ligand internalization is crucial for pyroptosis

**Fig 4:**
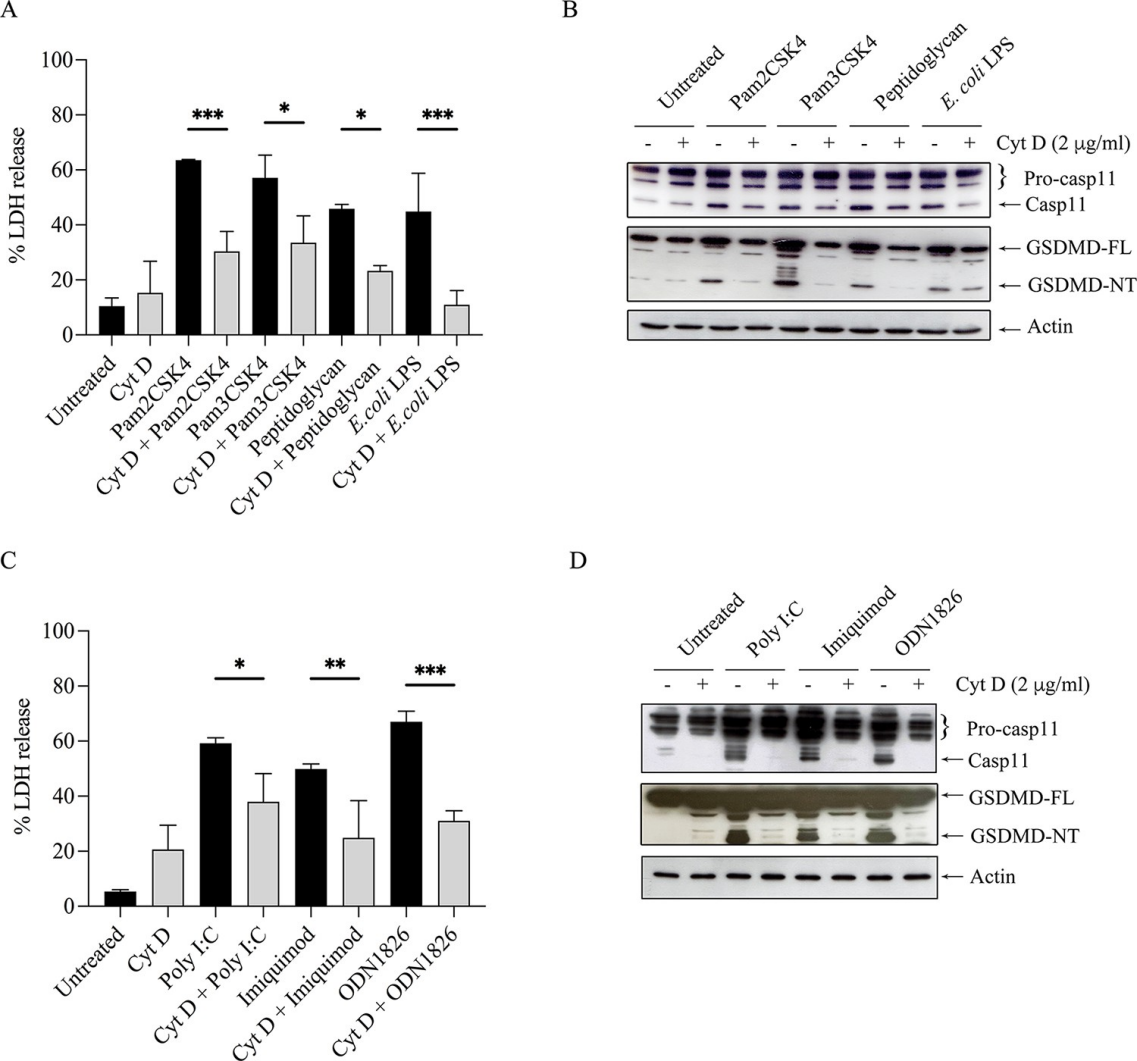
Cytochalasin D decreases pyroptosis in TLR ligand-activated Raw264.7 cells. Raw264.7 cells were pretreated with 2 μg/ml cytochalasin D (Cyt D) for 2 h before stimulation with TLR ligands for 18 h. (A, C) At the indicated time, the cultured supernatant of treated cells was collected and analyzed by the LDH assay. Data are means ± SEM from at least three independent experiments. All data were determined by one-way ANOVA followed by Tukey’s multiple comparison test. *p < 0.05, **p < 0.01, ***p < 0.001. (B, D) The cell lysates were prepared and analyzed by immunoblotting. Representative bands are shown from three independent experiments.

As internalization of TLR4 and TLR2 by endocytosis was shown to trigger signaling, endocytosis of TLRs may be crucial in TLR-mediated pyroptosis [26]. To test whether activation of pyroptosis was dependent on internalization of the TLR ligands, Raw264.7 cells were pre-treated with cytochalasin D, an endocytosis inhibitor, before TLR ligand treatment. As expected, this inhibitor reduced the expression of caspase-11 and GSDMD-NT in both surface and endosomal TLR-agonist-treated cells (Fig 4B and 4D). In addition, these results were also consistent with the markedly decreased LDH release in the presence of cytochalasin D (Fig 4A and 4C).

### Endosome acidification involves in pyroptosis

**Fig 5:**
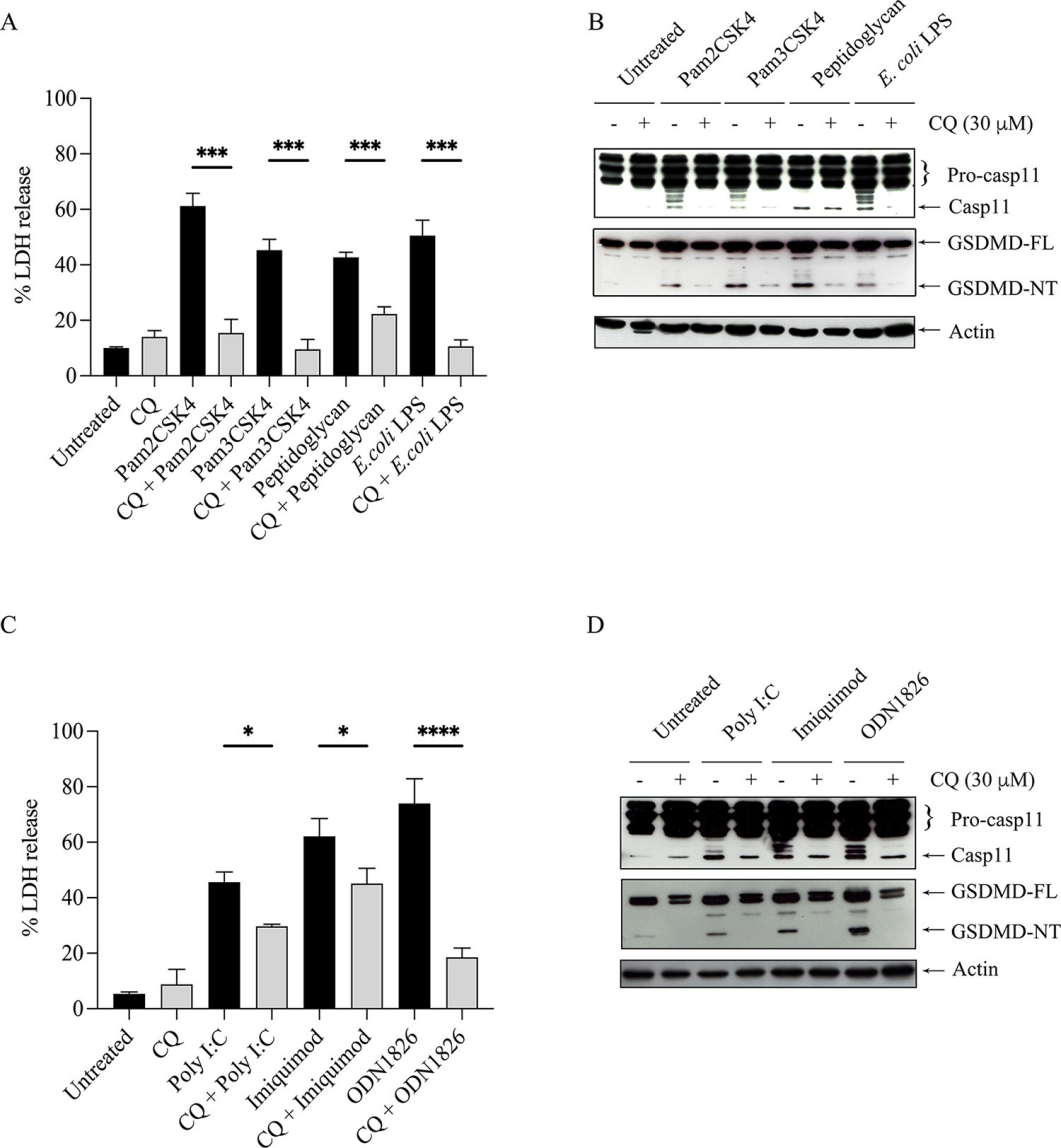
Chloroquine inhibits pyroptosis in TLR ligand-activated Raw264.7 cells. Raw264.7 cells were pretreated with 30 μM chloroquine (CQ) for 15 min before stimulation with TLR ligands for 18 h. (A, C) At the indicated time, the cultured supernatant of treated cells was collected and subjected to the LDH assay. Data are means ± SEM from at least three independent experiments. All data were determined by one-way ANOVA followed by Tukey’s multiple comparison test. *p < 0.05,***p < 0.001,****p < 0.0001. (B, D) The cell lysates were prepared and analyzed by immunoblotting. Representative bands are shown from three independent experiments.

Next, we asked whether endosome acidification was associated with TLR-agonist-induced pyroptosis. As shown in Fig 5A, in cells triggered by TLR2 and TLR4 ligands, chloroquine (CQ) markedly reduced LDH release and the cleavage of caspase-11 and GSDMD (Fig 5B). Similar results were also observed when the cells were stimulated with endosomal TLR ligands in the presence of CQ (Fig 5C and 5D). These results indicated that endosomal acidification is essential for surface and endosomal TLR-agonist-induced pyroptosis.

### ECDD-S16 inhibits lysosome acidification

**Fig 6:**
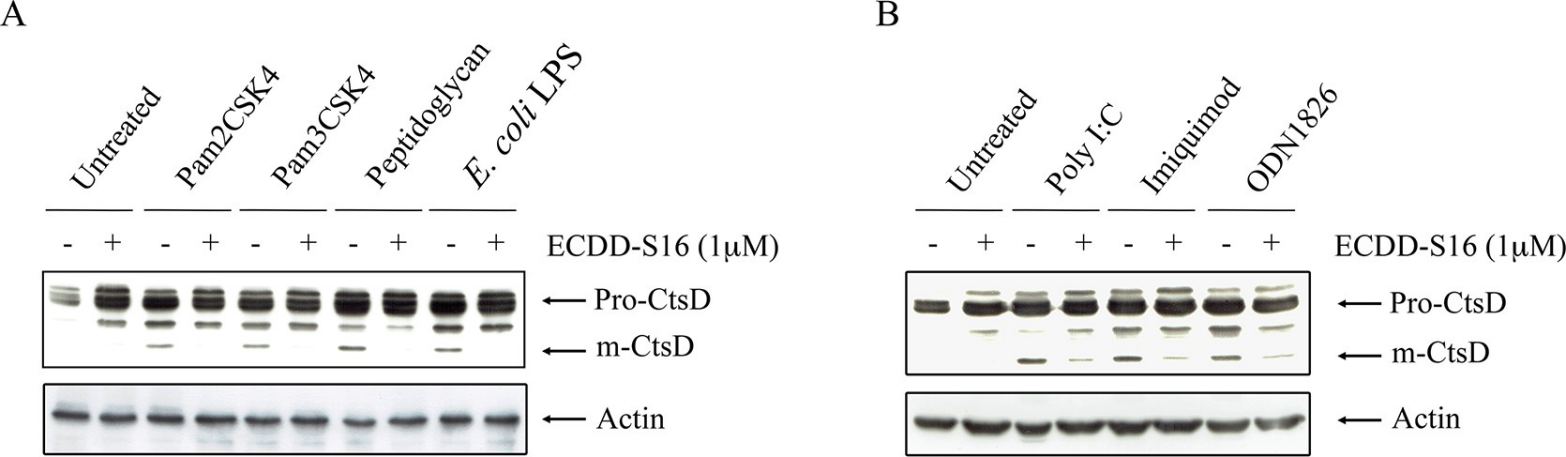
ECDD-S16 inhibits lysosome acidification by suppressing cathepsin D activation in TLR ligand-treated Raw264.7 cells. Raw264.7 cells were pretreated with ECDD-S16 (0.5 μM) for 1 h before stimulation with TLR ligands. After 18 h of activation, the cell lysates were collected and the expression of cathepsin D was determined by immunoblotting. Representative bands are shown from three independent experiments.

**Fig 7:**
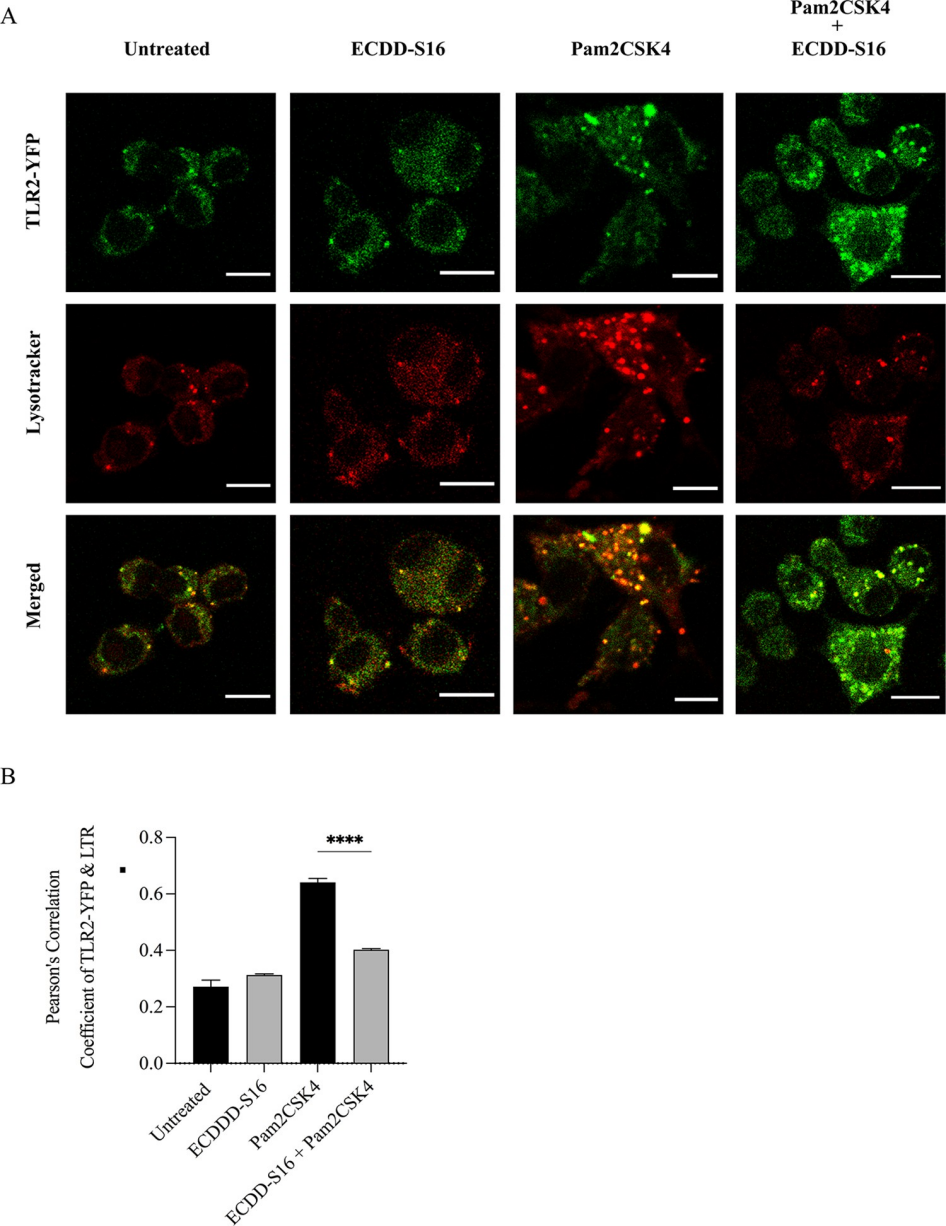
Pam2CSK4 induces internalization and trafficking of TLR2 into the endolysosomal compartment. Raw264.7 macrophages were transfected with TLR2-YFP (green) for 48 h. After that, the transfected cells were stimulated with Pam2CSK4 (1 μg/ml) for 1 h. Acidification of endosomes/lysosomes was then determined by adding the Lysotracker Red (LTR) dye to these cells in the presence or absence of ECDD-S16 and incubation was continued for 2 h. The treated cells were then fixed and processed for CLSM image analysis. (A) Confocal microscopy showing the colocalization of TLR2-YFP and LTR. Scale bar is 10 μm. (B) The co-localization of TLR2-YFP and LTR was quantified. All data were determined by one-way ANOVA followed by Tukey’s multiple comparison test. ****p < 0.0001.

Cathepsin D is a lysosomal acid hydrolase and a marker of lysosome [27]. Upon entering the acidic lysosomal compartment, the 44 amino acid N-terminal propeptide cleavage of cathepsin D occurs generating a 48 kDa intermediate active enzyme form. Therefore, we further investigated if the inhibition of pyroptosis by ECDD-S16 involves lysosome acidification in Raw264.7 cells activated with TLR ligands. As shown in Fig 6, ECDD-S16 reduced the amount of the mature form of cathepsin D (m-CtsD), indicating that ECDD-S16 interferes with lysosome acidification in TLR-ligand treated Raw264.7 cells. Moreover, we further investigated if ECDD-S16 can interfere with TLR-agonist-induced lysosomal acidification. The results revealed that ECDD-S16 significantly impaired the colocalization of TLR2-YFP and lysotracker (Fig 7), suggesting decreased the loss of acidity in cellular compartments after TLR2 internalization.

### ECDD-S16 impairs ROS production

**Fig 8:**
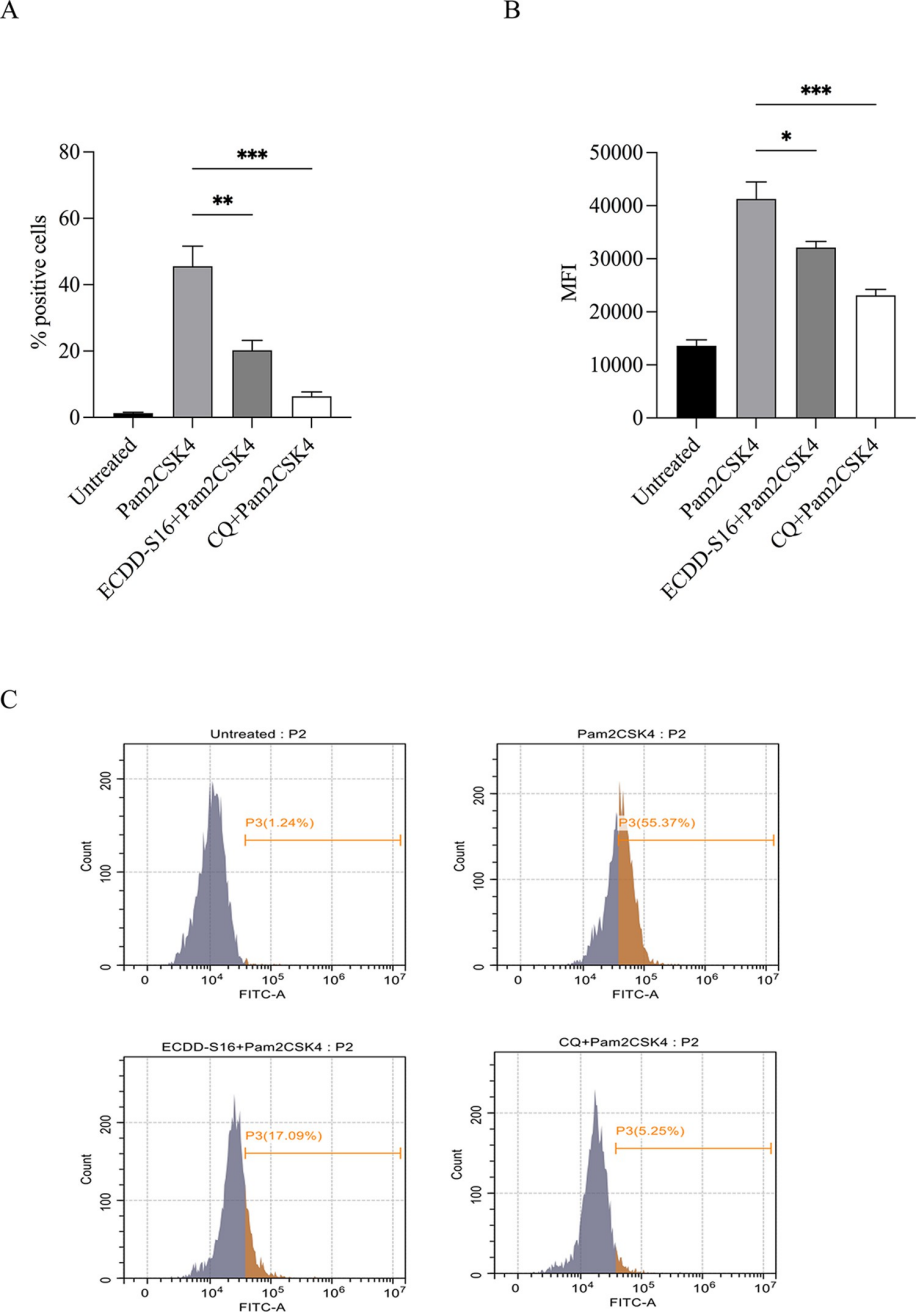
Effect of ECDD-S16 on Pam2CSK4-induced ROS production in Raw264.7 cells. Raw264.7 cells were pretreated with ECDD-S16 (0.5 μM) for 1 h or 30 μM chloroquine (CQ) for 15 min before stimulation with Pam2CSK4 (1 μg/ml) for 16 h. Then, the treated cells were washed and incubated with 2 μM of DCFH-DA for 20 min at 37°C. ROS production was analyzed by flow cytometry. The percentage of positive cells (A), mean fluorescent intensity (MFI) (B) and representative histograms of DCFH-DA (C) are shown. The results are presented as means ± SEM of three independent experiments. All data were determined by one-way ANOVA followed by Tukey’s multiple comparison test. *p < 0.05, **p < 0.01,***p < 0.001.

Given that ROS plays a role in inflammasome activation leading to pyroptosis [28], we sought to determine whether inhibition of endosome acidification by ECDD-S16 can interfere with ROS production. The results showed that Pam2CSK4 could stimulate ROS production in macrophages up to 55.37% (Fig 8A and 8C). However, in the presence of ECDD-S16, the level of intracellular ROS was significantly reduced to 17.09%. Consistently, chloroquine could also reduce ROS production (Fig 8A and 8C). Moreover, the mean fluorescent intensity was also analyzed (Fig 8B). Taken together, endosome acidification plays a role in ROS production in response to TLR ligand treatment. Inhibition of endosome acidification by ECDD-S16 interfers with ROS generation and may also decrease pyroptosis.

### Vacuolar ATPase, a potential ECDD-S16 binding target

**Fig 9:**
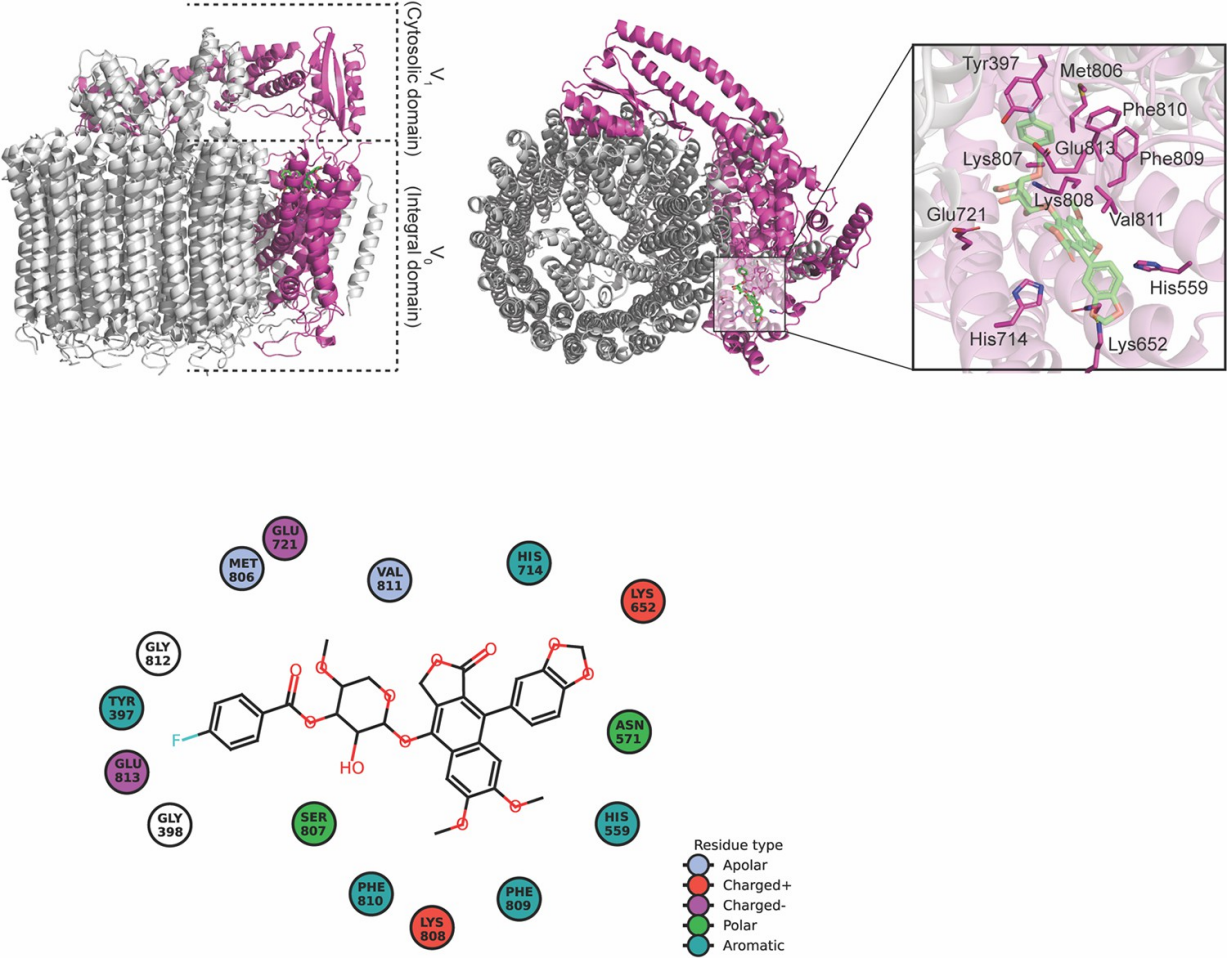
The illustration of V-ATPase and ECDD-S16 interaction. (A) Image depicts the binding between ECDD-S16 and V-ATPase V0 region. (B) A close-up view of the binding pocket between V-ATPase and ECDD-S16 illustrates the location of neighboring amino acids around ECDD-S16. (C) The two-dimensional diagram demonstrates the key amino acids stabilizing the binding between ECDD-S16 and V-ATPase.

Previously, the results showed that the ECDD-S16 downregulated the acidification of endosomes. We therefore hypothesized that ECDD-S16 might block the activity of V-ATPase, a key protein that regulates the H^+^ pump into the endosome/lysosome. By using the molecular docking, the results showed that ECDD-S16 possibly binds to the V0 region of the c-ring, the integral domain of V-ATPase, which plays a role in proton (H^+^) transport across the endosomal membrane (Fig 9A) with the binding energy of -7.5 kcal/mol. The close-up scheme illustrated that the predicted ECDD-S16 binding pocket contains non-polar amino acids and aromatic amino acids such as Val811, Gly812, Gly398, Phe809, Phe810, which help to promote the hydrophobic interaction (Fig 9B and 9C). From previous studies, the proton’s movement across the channel was shown to be dependent on glutamic acid, arginine and lysine. Our results demonstrated that Glu813, Glu712, Lys652 and Lys808 might be involved in H^+^ transportation. Especially, Lys652 could form the hydrogen bonding with ECDD-S16.

## Discussion

Pyroptosis has been known to be associated with innate immunity and infectious diseases. Recently, it has been demonstrated that activation of caspase-11/GSDMD is vital for *Brucella abortus* killing. Mice deficiency of GSDMD or caspase-11 were more susceptible to *B*. *abortus* infection [29]. In addition, the autophagic process has been impaired in *Gsdmd*-deficient mice infected with *Burkholderia cenopacia*, suggesting that GSDMD is involved in pyroptosis [30]. Besides infectious diseases, several evidences also demonstrated that pyroptosis plays a pivotal role in cancers such as melanoma and breast and lung cancers [31]. For example, GSDMD was found to be upregulated in non-small lung cancer (NSCLC), which promoted tumor metastasis [32]. Conversely, silencing GSDMD could inhibit transplanted tumor growth in mice [32]. Therefore, the regulation of tumor pyroptosis may be a target for tumor treatment.

Mechanistically, pyroptosis is driven by two main signaling pathways—one mediated by caspase-1 activation via inflammasome and the other by caspase-4/5 (human) or caspase-11 (mouse), which results in the cleavage of GSDMD into GSDM-NT and triggers pyroptotic cell death [13]. It was also shown that the binding of cytosolic LPS to caspase-11 leads to pyroptosis [13]. Interestingly, our data presence here showed that not only LPS but all TLR ligands could activate caspase-11 resulting in the cleavage of GSDMD and LDH release suggesting that all TLR ligands can activate caspase-11. In addition, ECDD-S16 not only inhibits cytokine production (Fig 2), but also attenuates LDH release in the Raw264.7 cells stimulated with both surface and endosomal TLR ligands (Fig 3). Moreover, both surface and endosomal TLR ligands can activate caspase-11 and GSDMD cleavage. Since IFN-β is known to play a pivotal role in controlling caspase-11 expression and activation [1, 10, 11], the decrease of caspase-11 activation by ECDD-S16 observed here may be due to the reduction of IFN-β. Interestingly, peptidoglycan and poly I:C failed to activate IFN-β production (Fig 2). However, both ligands can induce pyroptosis (Fig 3), similar to that of other TLR ligands which suggested the difference in pyroptosis pathway induced by these two ligands and the others. This point remains to be investigated.

Although surface TLRs such as TLR2 and TLR4 are expressed on the cell surface, internalization of these TLRs is also essential for signaling. TLR4 activates two signaling pathways, one on the surface and the second requires internalization into the endosome [33–35]. The LPS-induced internalization of TLR4 into the endosome, similar to endosomal TLRs, occurs via clathrin-independent and/or clathrin-dependent pathways [5, 36, 37]. By neutralizing the pH of the endosome with chloroquine, the production of the MyD88- and TRIF-dependent cytokines decreases, suggesting that endosome acidification is vital for cytokine production [34]. Similar to TLR4, cytokine production is decreased in TLR2-ligand activated cells in the presence of bafilomycin A1 (BafA1) [6]. Acidification also plays a significant role in endosomal TLR signaling. These receptors require receptor proteolysis, which occurs only at acidic pH in the endolysosome [38]. In our study, we demonstrated that by inhibiting internalization of the TLRs with cytochalasin D (Fig 4) and endosome acidification with chloroquine (Fig 5), pyroptosis induced by TLR ligands was decreased, suggesting both internalization and acidification are crucial for pyroptosis. We also demonstrated that ECDD-S16 could decrease the amount of the mature form of cathepsin D and reduce the colocalization of TLR2-YFP with lysotracker when the cells were treated with Pam2CSK4 (Fig 7).

ROS has been known as a signal mediator, which plays a crucial role in fighting against infection and is involved in the growth, differentiation, progression, and death of the cells. Generation of ROS is typically increased during inflammation response, followed by NF-κB activation leading to cytokine production [39]. This result is also consistent with our findings that inhibition of ROS production (Fig 8) by ECDD-S16 also attenuates cytokines production (Fig 2). Several studies also showed that ROS serves as an inflammasome activating signal [40–42]. ROS production has also been demonstrated to regulate caspase-11 expression and activation, leading to pyroptosis in *Citrobacter rodentium*-infected bone marrow-derived macrophages (BMDMs) [28]. In our study, we also showed the production of ROS was impaired in the presence of ECDD-S16 (Fig 8). Therefore, the inhibition of endosome acidification may affect ROS production, which leads to the inhibition of pyroptosis via caspase-11 and GSDMD activation.

Vacuolar ATPase, a hemichannel transmembrane protein, plays a role in proton pump across the membrane, causing acidification in many compartments such as endosomes, lysosomes and vesicles. Several studies showed that inhibition of V-ATPase is associated with human diseases due to the defect in the elimination of unuseful biomolecules of the cell [43]. Moreover, the inhibition of Vacuolar ATPase cytosolic region by Lactoferrin, a protein from milk, can interfere with the progression of cancer [44]. The acidified mechanism of V-ATPase occurs by pumping the proton across the membrane region (V0) via ATP hydrolysis by the cytosolic region (V1), thus allowing the proton transport unidirectionally from the cytosol to lumen [41]. Our study demonstrated that ECDD-S16 has a core structure similar to diphyllin, which was previously reported as a V-ATPase inhibitor [45, 46]. Our results also showed that ECDD-S16 can possibly bind to V-ATPase at the hemichannel, and thus may interrupt the carboxyl side chain of Glu813 and Glu712 (Fig 9). Furthermore, Lys652 and Lys808, located inside the luminal domain of V-ATPase hemichannel, may also prevent proton transportation across the membrane at V0 domain. This study showed that the disruption of V-ATPase by ECDD-S16 is crucial for ROS production, leading to pyroptosis induced by TLR ligands.

In conclusion, our finding demonstrated that ECDD-S16 can suppress pyroptosis/inflammation by interfering with endolysosome acidification. This compound might be a promising compound for treatment as an anti-inflammatory drug.

## Supporting information

S1 Raw images (PDF)
