# Supplementary figures and images for: ECDD-S16 targets vacuolar ATPase: A potential inhibitor compound for pyroptosis-induced inflammation
Source: PLoS One. 2023 Nov 27;18(11):e0292340. doi: 10.1371/journal.pone.0292340 (PMC10681236; doi:10.1371/journal.pone.0292340)

Fig. 3

B)

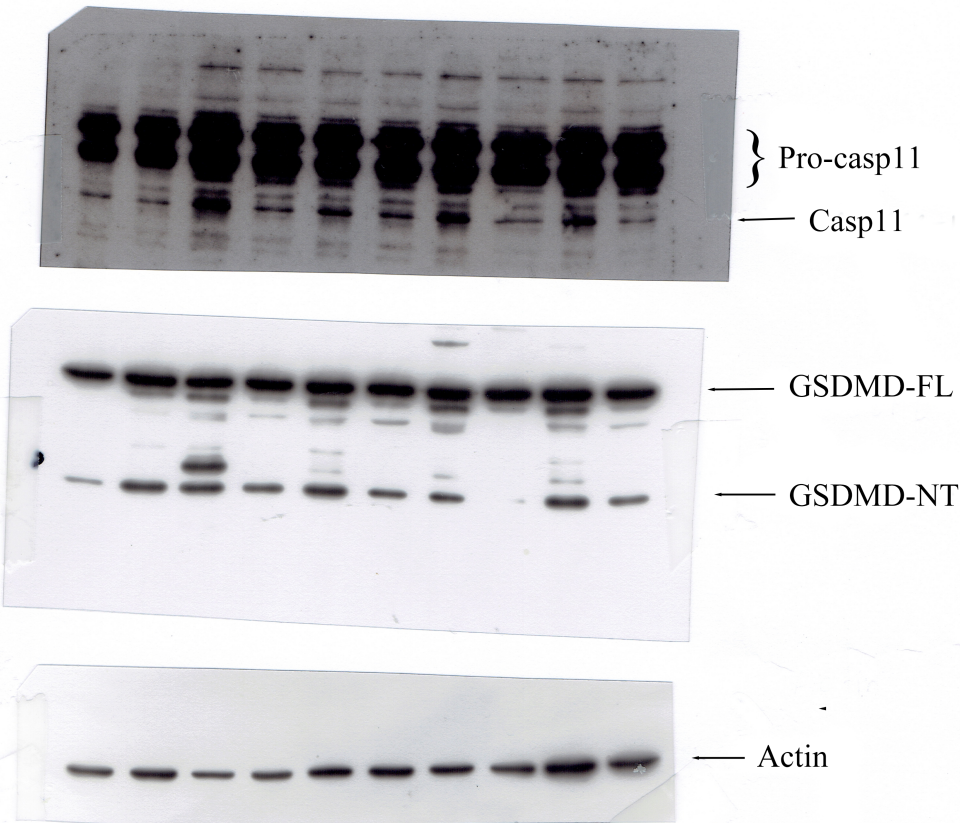

D)

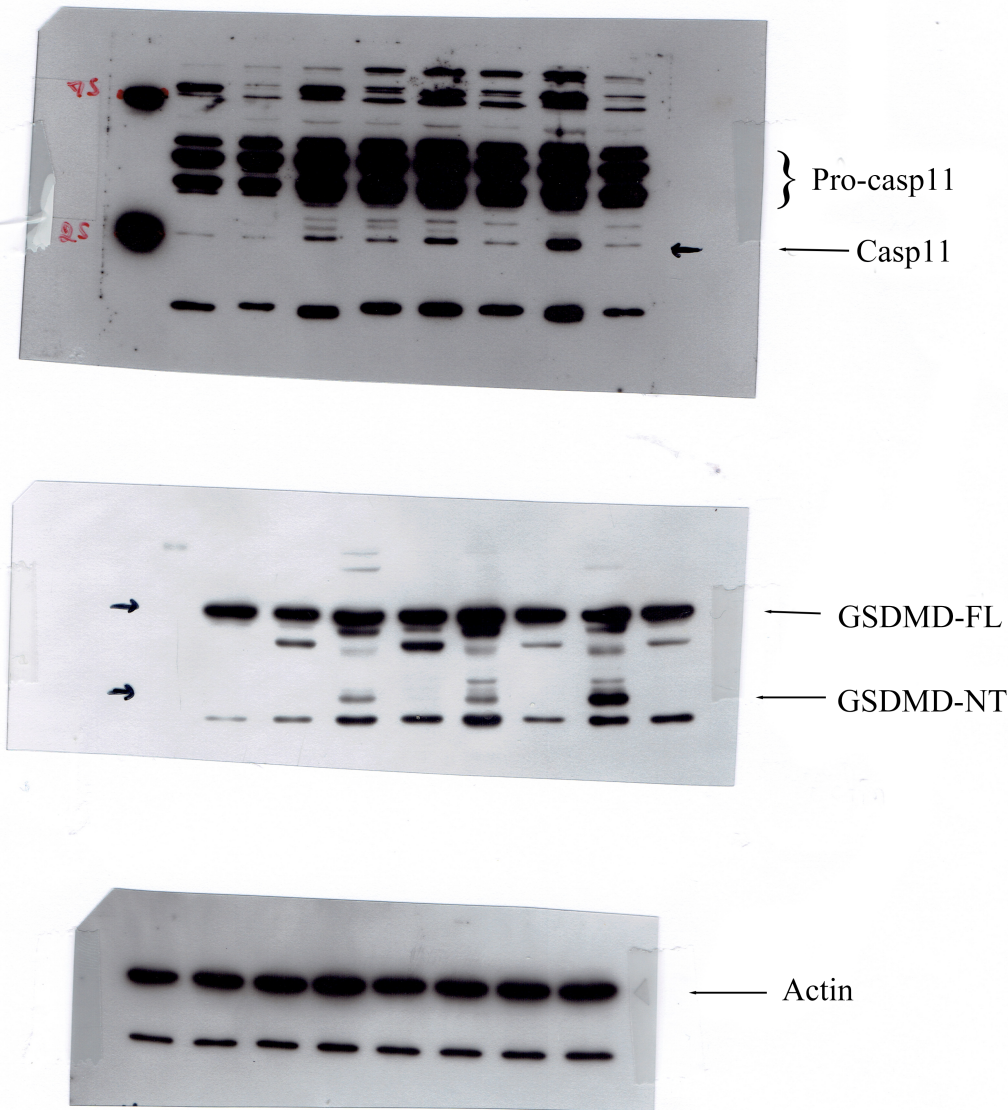

Fig. 4

B)

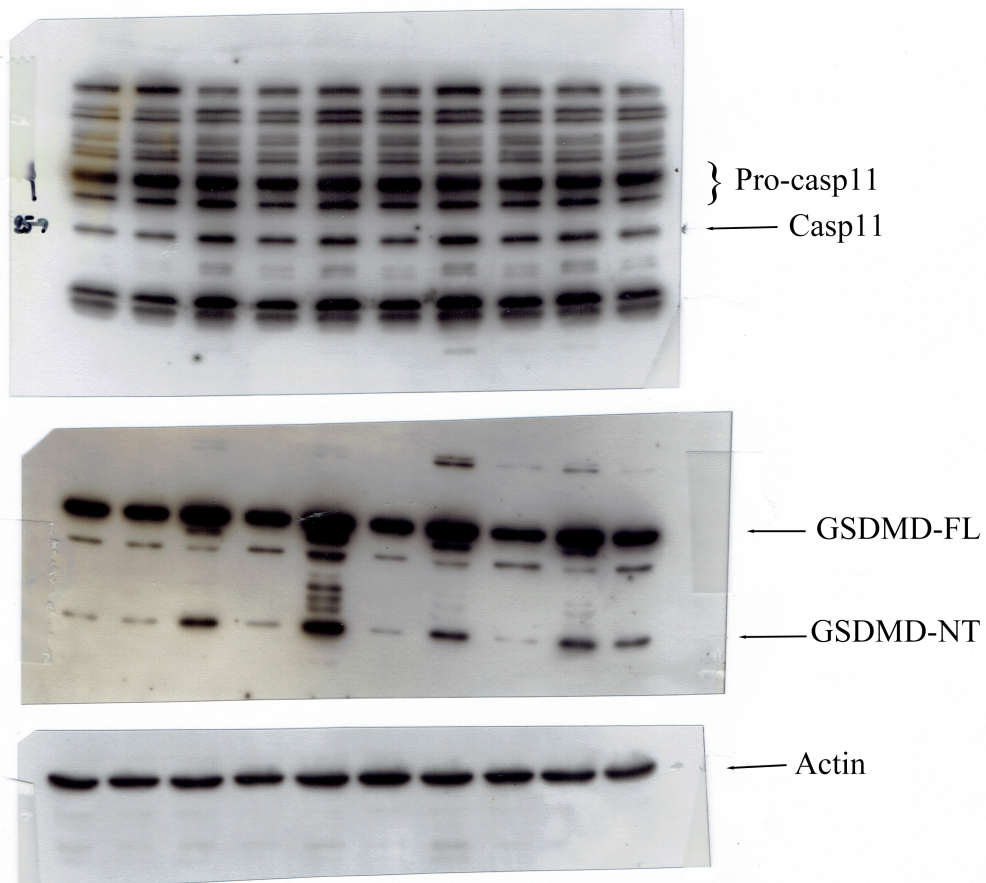

D)

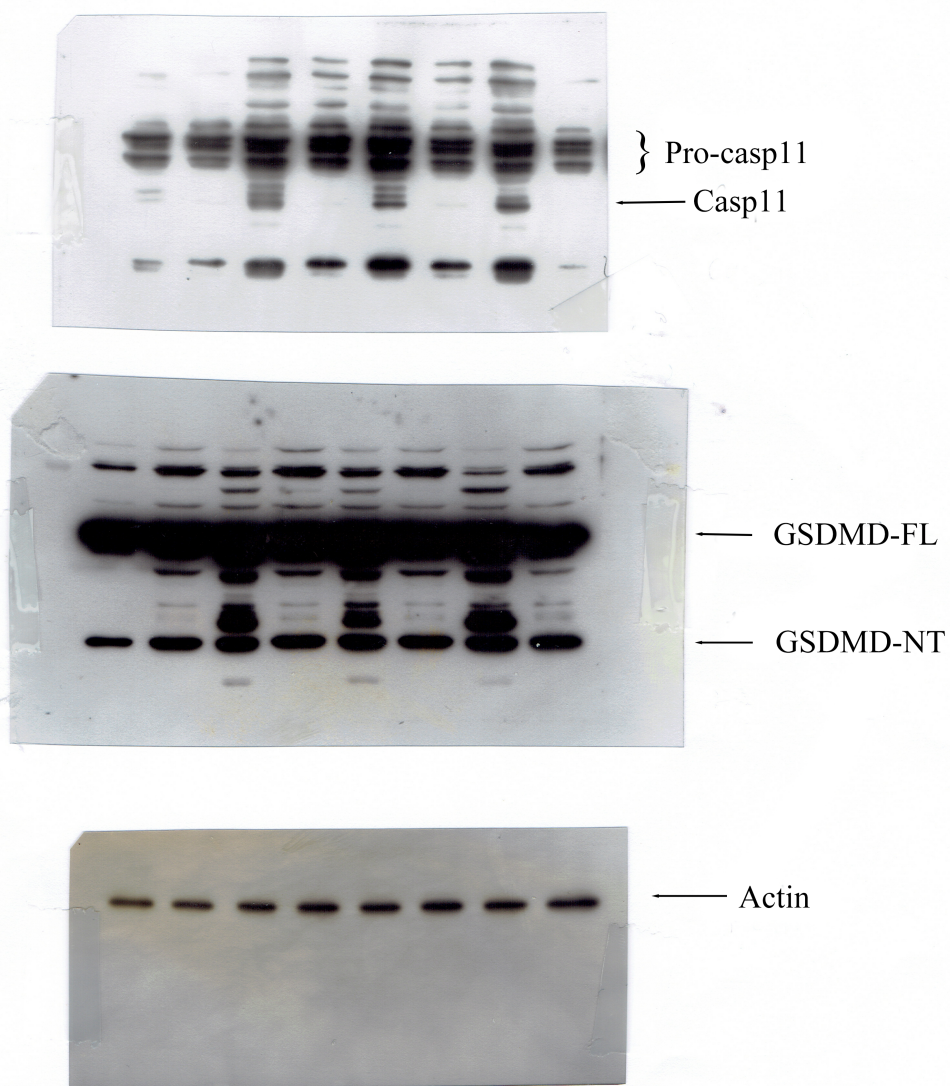

Fig. 5

B)

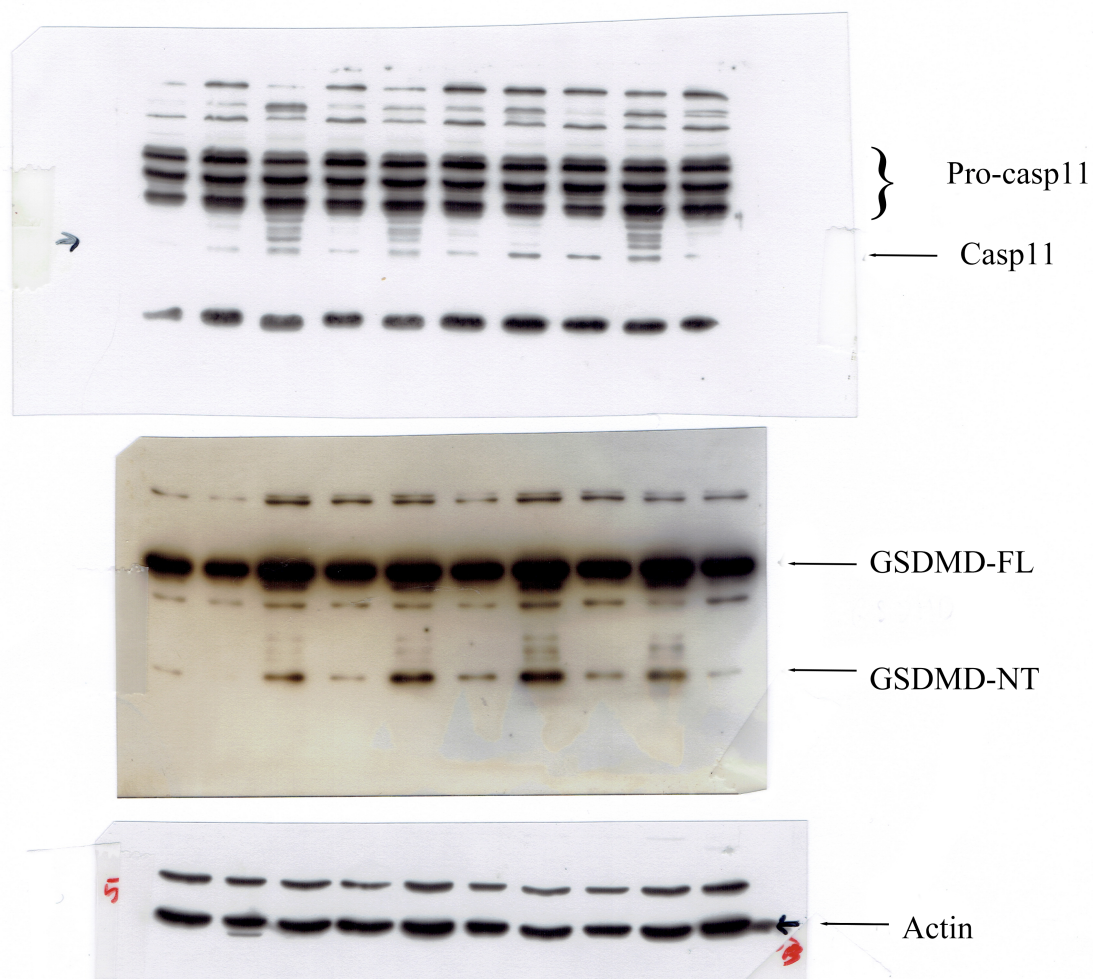

D)

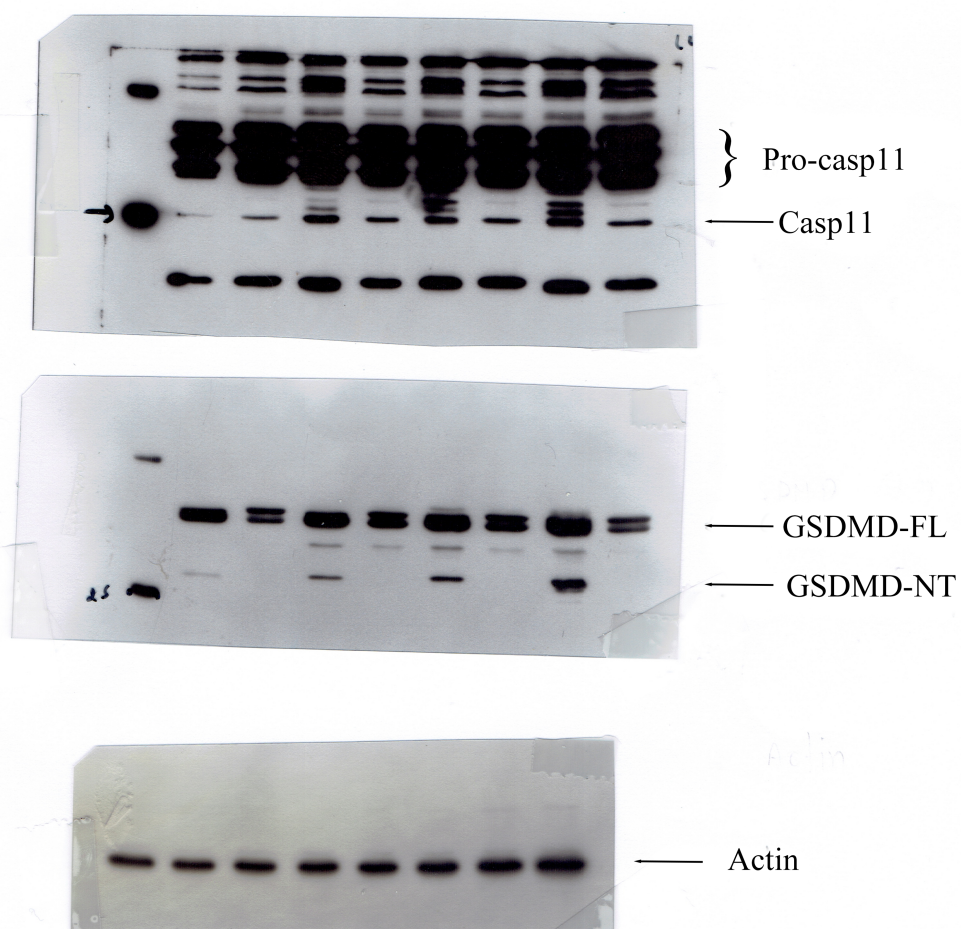

Fig. 6

A)

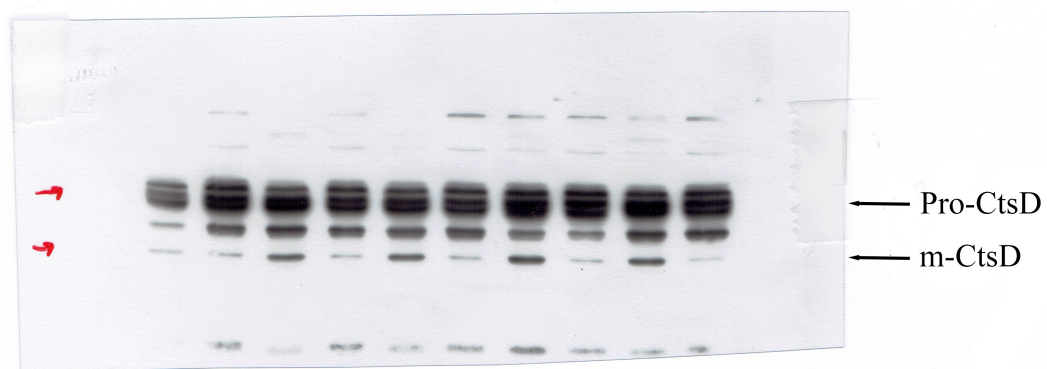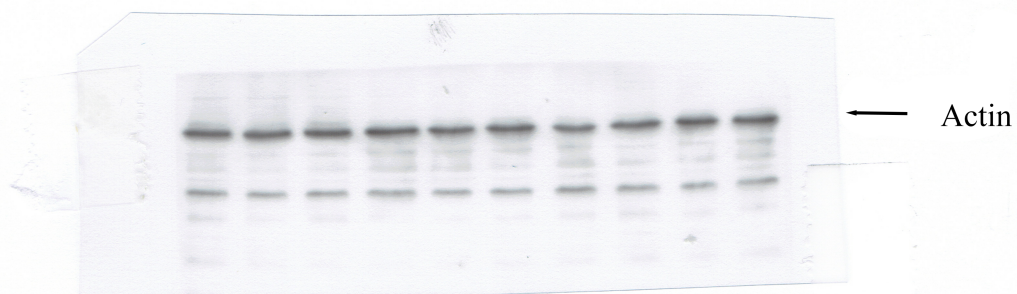

B)

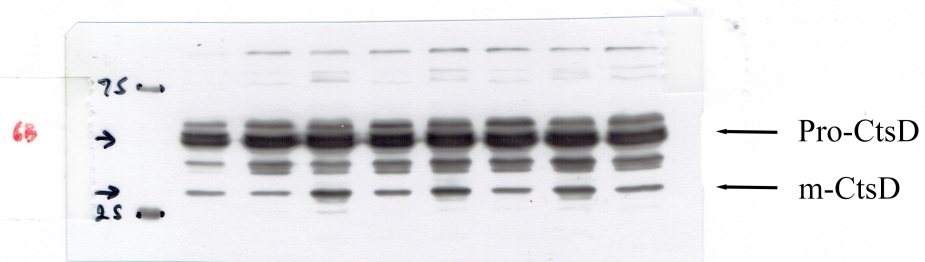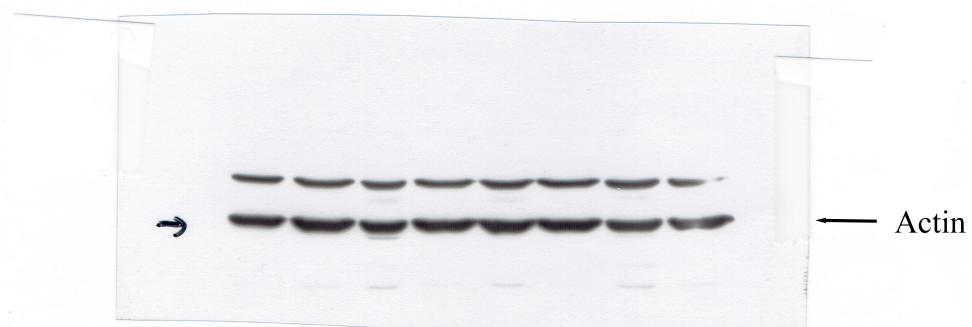

Supplement: S1 Raw images — (PDF) [file pone.0292340.s001.pdf]
